# Designing Trustworthy Product Recommendation Virtual Agents Operating Positive Emotion and Having Copious Amount of Knowledge

**DOI:** 10.3389/fpsyg.2019.00675

**Published:** 2019-04-02

**Authors:** Tetsuya Matsui, Seiji Yamada

**Affiliations:** ^1^Department of Computer and Information Science, Faculty of Science and Technology, Seikei University, Tokyo, Japan; ^2^Digital Content and Media Sciences Research Division, National Institute of Informatics, Tokyo, Japan; ^3^Department of Informatics, The Graduate University for Advanced Studies (SOKENDAI), Tokyo, Japan

**Keywords:** human-agent interaction, affective computing, anthropomorphic agent, virtual agent, PRVA, trustworthiness, emotional contagion

## Abstract

Anthropomorphic agents used in online-shopping need to be trusted by users so that users feel comfortable buying products. In this paper, we propose a model for designing trustworthy agents by assuming two factors of trust, that is, emotion and knowledgeableness perceived. Our hypothesis is that when a user feels happy and perceives an agent as being highly knowledgeable, a high level of trust results between the user and agent. We conducted four experiments with participants to verify this hypothesis by preparing transition operators utilizing emotional contagion and knowledgeable utterances. As a result, we verified that users' internal states transitioned as expected and that the two factors significantly influenced their trust states.

## 1. Introduction

In this paper, we suggest a model that increases the trustworthiness of a technological informant by designing its appearance and behavior. We focused on the trust between PRVAs and buyers. Agents that take part in online shopping and recommend products are called “product recommendation virtual agents” (PRVAs) (Qiu and Benbasat, 2009) and need to be trusted by customers in order for their buying motivation to increase. Lu et al. (2010) showed that trustworthiness perceived by consumers contributed to the buying motivation of consumers in e-commerce. In prior work, the design and behavior of PRVAs impacted the effects of their recommendations. Terada et al. inspected the effect of the appearance of PRVAs (Terada et al., 2015). It was revealed that appearance is a large factor in recommendation effects. It was found that a young female agent is one of the most effective PRVAs.

The trustworthiness of virtual agents as informants was studied in human-agent interaction. Virtual agents were perceived as real humans during interaction (Reeves and Nass, 1996); thus, the notion of trust that the agents displayed seemed to be near human.

Otherwise, virtual agents have the aspects of mechanical systems, and this fact affects their trustworthiness perceived by humans. Lucas et al. conducted an experiment in which participants were interviewed by virtual agents and were told that the agents were controlled by humans or automation. They showed that the humans were willing to report self-disclosure to the agents controlled by automation (Lucas et al., 2014). This result suggested that humans see virtual agents as computers; however, the agents have aspects of humans. Madhavan et al. (2006) showed that people lose their trust in computers more strongly than human advisers when they make a mistake. de Visser et al. showed that anthropomorphic virtual agents can reduce this effect. They showed that the anthropomorphic appearance reduces the disappointment humans feel when these agents did not to live up to the humans' expectations (de Visser et al., 2016). Thus, we concluded that virtual agents' trustworthiness has both human and computer aspects. The characteristics of virtual agents discovered in these pieces of research, that is, drawing out self-disclosure and reducing disappointment when the agents fail, seemed to be serviceable for recommending products.

Next is which virtual agent is trusted by users. Danovitch and Mills (2014) showed that children trust familiar virtual characters more than unfamiliar ones as informants. In the case of adults, more natural and recognizable virtual agents bring about a more positive impression, including trustworthiness (Hertzum et al., 2002). These pieces of research focused on the appearance of virtual agents. In our research, we focused on the behavior design of a trustworthy virtual agent. In other words, we aimed to construct trust between users and agents through interactions.

In the case of a robot, it was reported that the task performance of robots was the most important factor in robots' trustworthiness perceived by users (Hancock et al., 2011; Salem et al., 2015).

Rossi et al. investigated the magnitude of the robots' error perceived by users (Rossi et al., 2017a) and conducted experiments about how timing and error magnitude affected trustworthiness of a robot (Rossi et al., 2017b). The error magnitude seemed to be a factor of task performance.

Task performance seems to also be one factor in virtual agents' trustworthiness; however, many virtual agents do not work in the factories and at disaster sites that robots do. Thus, task performance seems to not be the most important factor in the case of virtual agents.

Much research has been conducted on forming rapport, that is, the state in a human and a virtual agent trust each other. Zhao et al. (2014) suggested that, to form rapport, we need long-term interaction with verbal and non-verbal cues. Gratch et al. (2007) showed that virtual agents that respond infrequently to humans form rapport with humans more smoothly than agents that respond frequently. Self-disclosure was also an effective method for creating trust and rapport. In the case of e-commerce via the web, Moon (2000) declared that intimate information exchange (self-disclosure) made consumers feel safe enough to reveal their information to computers. The validity of this method in virtual agents was demonstrated (Kang and Gratch, 2010; Kang et al., 2012). However, it took more time than conventional methods before making recommendations. Thus, we aimed at suggesting a method that creates trust immediately, without long interactions.

We suggest that trust depends on two kinds of parameters, users' emotion and knowledgeableness perceived by users'. Fogg (2002) stated that credibility seems mainly to be constructed by “trustworthiness” and “expertise” in the area of psychology. “Trustworthiness” is based on whether the truster feels that the trustee is fair and honest. This may be affected by emotion. “Expertise” is based on a high level of knowledge, skill, and experience. This is equal to knowledgeableness perceived. Fogg stated these two factors were important for persuasion via computer.

Many prior studies showed that emotional state was one of the important parameters for judging the trustworthiness of partners or informants. Dunn and Schweitzer (2005) showed that people tended to trust unfamiliar people when they were happy. Druckman and McDermott (2008) showed that people made risky choices on the basis of emotion. Also, Dong et al. (2014) showed that people tend to trust partners that have a positive expression. These pieces of research showed that a positive emotion is important for building trust. In psychology and cognitive science, Lang (1995's) dimensional emotional model is widely used. We used only the valence axis, positive or negative, in this model because the above prior pieces of work showed that a truster's positive or negative state affected the perception of trustworthiness.

The trustworthiness of informants seems to be partly based on an informant's knowledgeableness. It was shown that informants that provided diverse examples were more trusted than informants that provided non-diverse ones (Landrum et al., 2015). This means that informants that have a more copious amount of knowledge were trusted. Adults tend to trust technological informants more than humans (Noles et al., 2016). This fact seems to be caused by the expectation that technological informants contain a lot of correct information. In the case of virtual agents, knowledgeableness seemed to be mainly judged by appearance. It was shown that an agent that looked more intelligent was more trusted by users (Geven et al., 2006). We aimed to make an anthropomorphic agent trustworthy by making it express that it is knowledgeable.

## 2. Trust Behavior Transition Model and Transition Operators

### 2.1. Trust Behavior Transition Model

In this study, we aimed to construct a model of a user's trust behavior transitions operated by a virtual agent's state transitions. In mental model theory, users update their model of a computer or agent after each output (Kaptelinin, 1996). In this work, we aimed to make users update their mental model of the trustworthiness of PRVAs.

**Figure 1:**
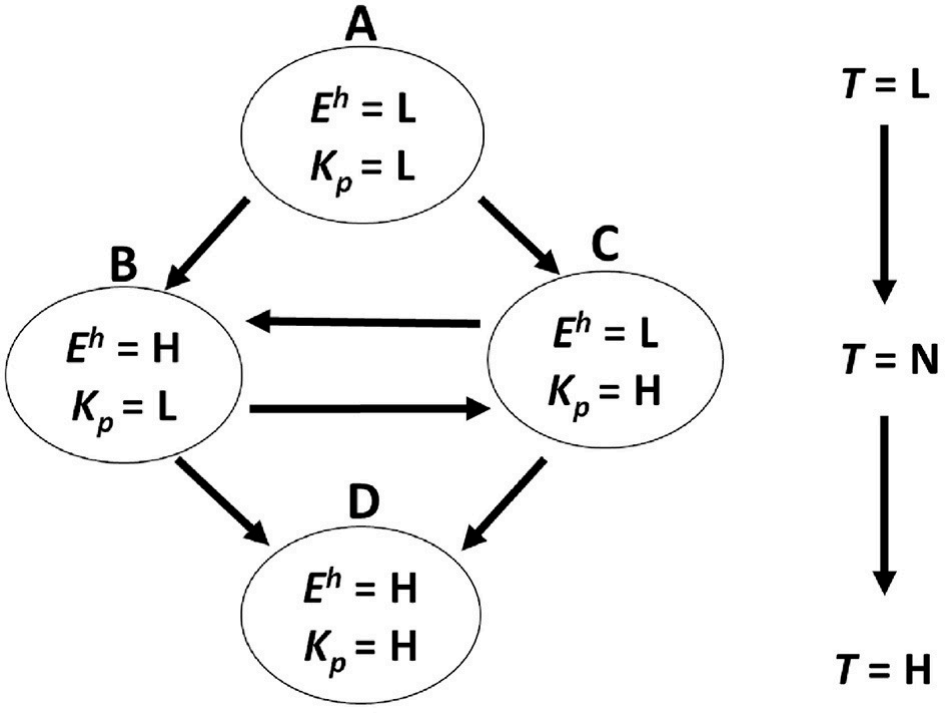
Transition model of user trust behavior.

Figure 1 shows a model of the transition in the internal trust behavior of users that we propose. From the many prior pieces of work (Dunn and Schweitzer, 2005; Geven et al., 2006; Druckman and McDermott, 2008; Dong et al., 2014; Landrum et al., 2015; Noles et al., 2016) that were cited in the introduction, we introduce “emotion” and “knowledgeableness perceived” as important factors that influence a user's trust state on the basis of the above discussion. In this model, we describe a user's internal state by using two parameters, < *E*^*h*^, *K*^*a*^>. *E*^*h*^ means the users' emotion, and *K*^*p*^ means the agents' knowledgeableness perceived by the users. We defined these two parameters by using the descriptions “L (Low)” or “H (High).” *E*^*h*^ means whether a user feels a positive emotion (H) or not (L). *K*^*p*^ means whether a user feels an agent is knowledgeable (H) or not (L). First, if *E*^*h*^ and *K*^*p*^ are both L, we describe the internal state as < *E*^*h*^ = L, *K*^*a*^ = L>. Also, if *E*^*h*^ and *K*^*p*^ transition to H, we describe the internal state as < *E*^*h*^ = H, *K*^*a*^ = L> or < *E*^*h*^ = L, *K*^*a*^ = H>. Finally, if both parameters transition to H, we describe the state as < *E*^*h*^ = H, *K*^*a*^ = H>. Also, we describe a user trust state as *T*. We aimed to inspect the *T* value for each state. We defined the trust level by using the descriptions “L (Low),” “N (Neutral),” and “H (High).” In our hypothesis, the trust state is L when the internal state is < *E*^*h*^ = L, *K*^*a*^ = L>. When the internal state is < *E*^*h*^ = H, *K*^*a*^ = L> or < *E*^*h*^ = L, *K*^*a*^ = H>, the trust state is N. Also, when the internal state is < *E*^*h*^ = H, *K*^*a*^ = H>, the trust state is H. L and H is relative value in an internal state, not absolute value. Thus this model can be used regardless of the users' mood and emotion in the first state.

It was not clear whether a positive emotion and knowledgeableness have a logical conjunction on the trustworthiness of PRVAs or not, so we focused on this conjunction. These trust states, the transition model, and the following transition operators for a PRVA are our original work.

### 2.2. Transition Operators

We introduce the notions of emotion and knowledgeableness transition operators for this research. These are executed when a PRVA is making recommendations that are expected to cause internal behavior transitions. We defined the emotion transition operators as a PRVA's smile and cute gestures and the knowledgeableness transition operators as a PRVA's technical and detailed knowledge.

Human emotion can be transmitted to other people through facial expressions, voice, or body movement. This is called *emotional contagion* (Hatfield et al., 1994), and it can occur between a virtual agent and a human through facial expressions (Tsai et al., 2012). This study was conducted via Amazon Mechanical Turk, the crowdsourcing service containing the participants in all over the world. Thus, the result of study has cultural generality.

These studies showed that a smiling agent causes users to feel happy. In the case of robots, Si and McDaniel (2016) showed that robots expressing a relaxed facial expression and body movement caused users to perceive the robots as friendly and trustworthy. This result could be caused by emotional contagion. Kose-Bagci et al. showed that drumming robot's head gesture increased the users' subjective fun (Kose-Bagci et al., 2009) and motivation of interaction (Kose-Bagci et al., 2010). These results showed that the robots' gesture affected the users' internal state. Kose et al. (2012) showed that the virtual agents' gesture and humans' gesture had the same effect in sign language tutoring. This result shows that executing the humans' gesture on the virtual agent is effective for affecting to the users' internal state.

**Figure 2:**
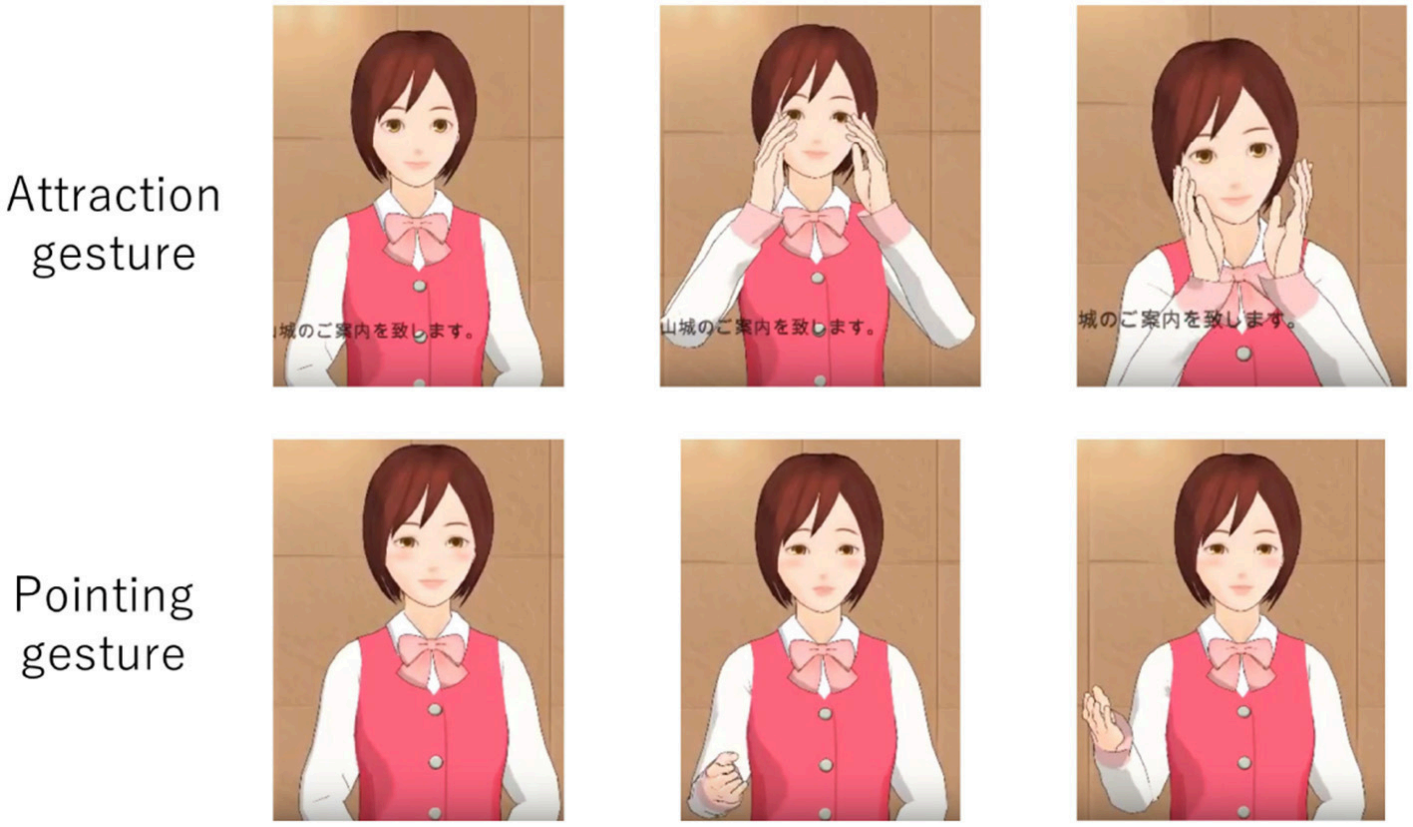
Gestures that we executed.

Thus, we aimed to affect a user's emotional state *E*^*h*^ with a PRVA through facial expressions and hand gestures in order to make the user trust the agent more strongly. We executed two kinds of hand gestures to bring about emotional contagion. The first gesture is an “attractive gesture,” in which the PRVA gestures its hands toward the mouse. The second gesture is a “pointing gesture,” in which the PRVA points out images. Figure 2 shows these gestures. Hence, we could utilize emotional contagion as a transition operator. User emotion becomes correspondent with agent emotion when emotional contagion is executed.

Also, we needed a transition operator for the other factor, knowledgeableness perceived *K*^*p*^. An agent's knowledgeableness is expressed through technical and detailed knowledge. Knowledgeableness perceived in a user state corresponds to knowledgeableness in an agent. If an agent looks knowledgeable, the user perceives it as being knowledgeable. We implemented concrete transition operators for emotion and knowledgeableness perceived in experiments and conducted the experiments with on-line shopping to verify this model.

## 3. Materials and Methods

We conducted four experiments with participants to verify our model. All experiments were conducted with the same implementations and same procedure. In all experiments, R1 means the recommendation that was shown to the participants first, and R2 means the recommendation that was shown to the participants second.

### 3.1. Experiment Design

All experiments were conducted on-line. All participants were recruited through Yahoo crowdsourcing^1^. The participants received 25 yen (about 0.22 dollars) as a reward. We conducted all experiments in September 2017. Detailed data on the participants are shown in sections for each experiment. All participants provided informed consent by clicking submit button, and the study design was approved by an research ethics committee in National Institute of Informatics.

The experiments took about 10 min for each participant. The participants were asked to watch movies in which a PRVA recommended a package tour to Japanese castles. The PRVA was executed with MMDAgent^2^, and the agent's character was “Mei,” a free model of MMDAgent distributed by the Nagoya Institute of Technology. We used VOCELOID+ Yuzuki Yukari EX^3^, which is text to speech software, for smooth utterances.

The PRVA recommended two package tours to two castles, and these recommendations are indicated by R1 and R2. The PRVA recommended package tours to Inuyama Castle and Gifu Castle. Both castles were built in the Japanese Middle Ages and have castle towers.

Neither of these castles are a World Heritage Site, and there seems to be no difference in the preferences of Japanese people between these two castles.

In all experiments, the PRVA recommended Inuyama Castle for R1 and Gifu Castle for R2 for a half of the participants. For the other half, the PRVA recommended Gifu Castle for R1 and Inuyama Castle for R2. This was for counterbalance.

**Figure 3:**
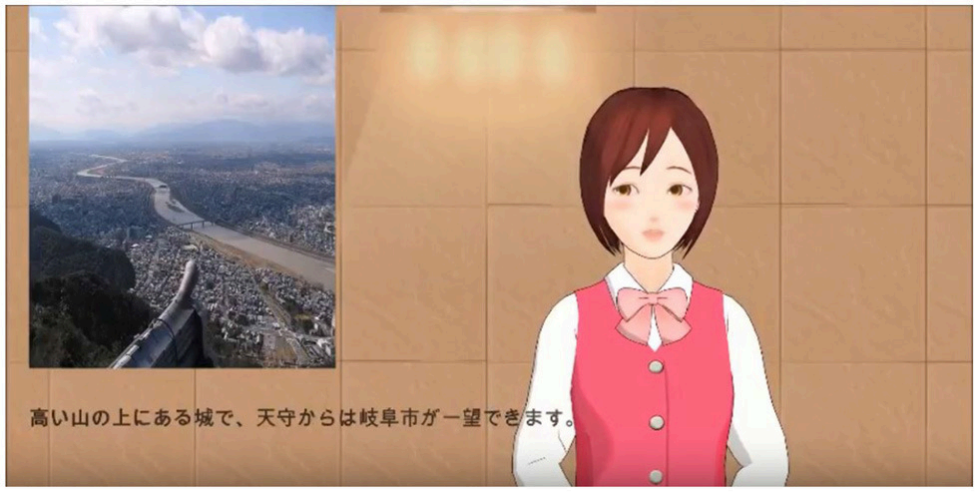
Snapshot from movie we used in experiment. The landscape image in this snapshot is licensed under the Creative Commons Attribution 2.5.

Figure 3 shows a snapshot from the movies. The transition operators executed by the agent for each recommendation are as follows. In experiment 1, no transition operator was executed for R1, and the emotion transition operators were executed for R2.

In experiment 2, no transition operator was executed for R1, and the knowledge transition operators were executed for R2. In experiment 3, the emotion transition operators were executed for R1, and the knowledge transition operators were also executed for R2. In experiment 4, the knowledge transition operators were executed for R1, and the emotion transition operators were also executed for R2. Some part of movies are shown as a Supplementary Material (Video 1).

**Figure 4:**
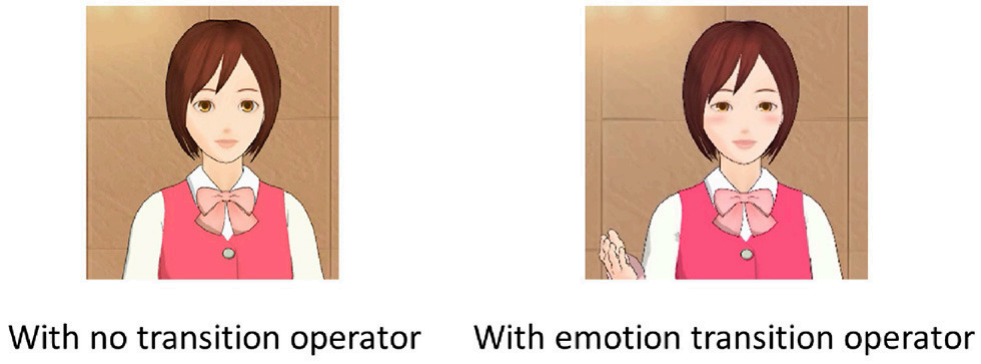
Snapshot of agent with and without emotion transition operators (smiling and gestures).

Figure 4 shows movie snapshots showing the agent without the emotion transition operators and with the operators. Table 1 shows which operators were executed for each recommendation in each experiment. Table 2 shows examples of speech that recommended a trip to Gifu Castle without the knowledge transition operators and with the operators. With the knowledge transition operators, the PRVA explained historical episodes and gave details on fees.

**Table 1:** Transition operators executed for each recommendation.

**Experiment**	**Recommendation 1**	**Recommendation 2**
Exp. 1	No operators	Emotion
Exp. 2	No operators	Knowledgeableness
Exp. 3	Emotion	Knowledgeableness and emotion
Exp. 4	Knowledgeableness	Knowledgeableness and emotion

**Table 2:** Recommendation text for Gifu Castle.

Without knowledge transition operators	Well, I recommend that you take a trip to
	a great sightseeing spot, Gifu Castle.
	Gifu Castle stands in Gifu
	City, Gifu Prefecture.
	This is the symbol of Gifu.
	You can feel special like a lord in this castle.
	Let's imagine who lived in this castle in olden days.
	We are selling a package tour to this castle,
	which is only the admission fee to the castle.
	However, the package tour also contains
	travel expenses and lunch.
	Don't miss it.
With knowledge transition operators	Well, I recommend that you take a trip to
	a great sightseeing spot, Gifu Castle.
	Gifu Castle stands in Gifu
	City, Gifu Prefecture.
	This castle stands on a mountain, so we can
	get a nice view from it.
	During the Warring State Period, the Saito family
	lived in the castle and reigned over the prefecture.
	Saito Dosan, the father-in-law of Oda Nobunaga,
	is the most famous member of the family.
	We are currently selling a package tour to this
	castle for only 21,060 yen, which is only the
	admission fee to the castle.
	However, the package tour also contains
	travel expenses and lunch.
	If you miss this chance, you'll surely
	regret it.

### 3.2. Questionnaires

Participants were each asked sets of questions after each recommendation was made. Thus, in total, they answered two sets of questions in one trial. The sets of questions were constructed with Wheeless's Interpersonal Solidarity Scale (ISS) and the Positive and Negative Affect Schedule (PANAS), a knowledgeableness scale. Wheeless's ISS is a scale for measuring the solidarity and trustworthiness of a particular person (Wheeless, 1978). We used this scale to measure the PRVA's trustworthiness that the participants perceived. This scale contains 20 questions. The participants were asked to answer these questions on a seven-point Likert scale. We calculated all scores and used the average as the Interpersonal Solidarity Score, the score of trust.

The Positive and Negative Affect Schedule (PANAS) is a scale that is used to measure a person's affect (Watson et al., 1988). This scale is based on the premise that affect is constructed of positive and negative affect. We used this scale to measure the participants' positive emotion. We used 16 questions, and the participants were asked to answer them on a six-point Likert scale. We used the sum of the score of eight questions related to positive affect as the Positive Affect Score for the score of positive emotion.

We constructed an original scale to measure knowledgeableness perceived, the knowledgeableness scale. This was constructed with these five questions shown in Table 3.

**Table 3:** Questions for knowledgeableness scale.

Q1	Do you feel that this agent is intelligent?
Q2	Do you feel that this agent has a copious amount of knowledge?
Q3	Do you feel that this agent has technical knowledge?
Q4	Do you feel that this agent has correct knowledge?
Q5	Do you feel that this agent is wise?

The participants were asked to answer these questions on a seven-point Likert scale. We defined the average of the score of these questions as the Knowledgeableness Score, the score of knowledgeableness perceived.

### 3.3. Statistical Procedure

For each experiment, we conducted a Wilcoxon signed-rank test between R1 and R2. The Wilcoxon signed-rank test is a non-parametric test that is widely used to compare two data sets and focuses on the transition between paired data (Gibbons and Chakraborti, 2011). This was the most suitable test for verifying the participants' internal state transition between the two recommendations. For the score on the PANAS and Knowledgeableness Scale, we defined the internal state (emotion and knowledgeableness perceived) transition as occurring when there was significant difference between each recommendation. We defined *E*^*h*^, *K*^*a*^, and *T* as L before the experiments. If the state was L before the recommendation and the score increased significantly after the recommendation, we defined the state as transitioning to H. If the state was H before the recommendation and the score decreased significantly after the recommendation, we defined the state as transitioning to L. Also, for the score on the ISS, we defined a trust state transition as having occurred when there was a significant difference. If the state was L before the recommendation and the score increased significantly after the recommendation, we defined the state as transitioning to N. Also, if the state was N before the recommendation and the score increased significantly after the recommendation, we defined the state as transitioning to H.

In this paper, we use the terms increased pair and decreased pair. Increased pair means the number of participants whose score increased between the two recommendations. Decreased pair means the number of participants whose score decreased between the two recommendations. They were the most important parameters in the Wilcoxon signed-rank test.

Each participants answered in total 119 questions in one trial. If a participant answered with the same number 20 times in a row, we excluded that participant as noise.

## 4. Result

### 4.1. Experiment 1: R1, No Transition Operator; R2, Emotion Transition Operators

In the first experiment, the PRVA made recommendations without any transition operators for R1 and also made recommendations with emotion transition operators for R2 (see also Table 1). We conducted this experiment with the aim of making the participants' state transition from state A to state B in Figure 1.

We recruited 219 Japanese participants for experiment 1, and 178 remained after noise exclusion. There were 112 males and 66 females, and they were aged between 22 and 74, for an average of 40.7 (*SD* = 8.4).

Table 4 shows the result of experiment 1. We conducted a Wilcoxon signed-rank test for these data. The Positive Affect Score (PA) significantly increased between the two recommendations (*p* < 0.01). The score on the Knowledgeableness Scale (KS) significantly decreased (*p* < 0.01). The score on the ISS significantly increased (*p* < 0.01).

**Table 4:** Result of experiment 1.

**Scale**	**R1 average (SD)**	**R2 average (SD)**	**Increased pair**	**Decreased pair**	***p***
PA	19.76 (6.17)	20.89 (7.27)	86	70	0.0052
KS	4.35 (1.06)	4.04 (1.23)	57	89	0.0001
ISS	60.67 (17.50)	67.17 (19.30)	112	58	0.0000

We conducted the same test for the result of female participants. As a result, PA non-significantly increased (*p* = 0.095), KS significantly decreased (*p* = 0.001) and ISS significantly increased (*p* = 0.003). Also, we conducted the same test for the result of male participants. As a result, PA significantly increased (*p* = 0.029), KS non-significantly decreased (*p* = 0.101) and ISS significantly increased (*p* = 0.000).

### 4.2. Experiment 2: R1, No Transition Operator; R2, Knowledgeableness Transition Operators

In experiment 2, the PRVA made recommendations without any transition operators for R1 and also made recommendations with knowledgeableness transition operators for R2. We conducted this experiment with the aim of making the participants' state transition from state A to state C in Figure 1.

We recruited 249 Japanese participants for experiment 2, and 209 remained after noise exclusion. There were 104 males and 105 females, and they were aged between 20 and 68, for an average of 42.2 (*SD* = 9.5).

Table 5 shows the result of experiment 2. We conducted a Wilcoxon signed-rank test for these data. For the PA, there was no significant difference. The KS significantly increased (*p* < 0.01). The ISS significantly increased (*p* < 0.05).

**Table 5:** Result of experiment 2.

**Scale**	**R1 average (SD)**	**R2 average (SD)**	**Increased pair**	**Decreased pair**	***p***
PA	19.06 (6.56)	18.73 (6.82)	73	96	0.1041
KS	4.21 (1.02)	4.42 (1.13)	108	48	0.0001
ISS	58.52 (17.40)	60.61 (17.67)	112	80	0.0105

We conducted the same test for the result of female participants. As a result, PA non-significantly decreased (*p* = 0.354), KS significantly increased (*p* = 0.001) and ISS significantly increased (*p* = 0.026). Also, we conducted the same test for the result of male participants. As a result, PA non-significantly decreased (*p* = 0.170), KS significantly increased (*p* = 0.011) and ISS non-significantly increased (*p* = 0.166).

### 4.3. Experiment 3: R1, Emotion Transition Operator; R2, Emotion and Knowledgeableness Transition Operators

In experiment 3, the PRVA made recommendations with emotion transition operators for R1 and also made recommendations with emotion and knowledgeableness transition operators for R2. We conducted this experiment with the aim of making the participants' state transition from state B to state D in Figure 1.

We recruited 255 Japanese participants for experiment 3, and 202 participants remained after noise exclusion. There were 100 males and 102 females, and they were aged between 19 and 75, for an average of 41.2 (*SD* = 9.7).

Table 6 shows the result of experiment 3. We conducted a Wilcoxon signed-rank test for these data. For the PA, there was no significant difference. The KS significantly increased (*p* < 0.05). The ISS significantly increased (*p* < 0.01).

**Table 6:** Result of experiment 3.

**Scale**	**R1 average (SD)**	**R2 average (SD)**	**Increased pair**	**Decreased pair**	***p***
PA	18.82 (5.89)	18.84 (6.58)	77	94	0.7932
KS	4.12 (1.15)	4.25 (1.20)	94	63	0.0154
ISS	58.13 (17.17)	62.90 (18.58)	118	76	0.0002

We conducted the same test for the result of female participants. As a result, PA non-significantly increased (*p* = 0.558), KS non-significantly increased (*p* = 0.085) and ISS significantly increased (*p* = 0.018). Also, we conducted the same test for the result of male participants. As a result, PA non-significantly increased (*p* = 0.998), KS non-significantly increased (*p* = 0.091) and ISS significantly increased (*p* = 0.002).

### 4.4. Experiment 4: R1, Knowledgeableness Transition Operator; R2, Emotion and Knowledgeableness Transition Operators

In experiment 4, the PRVA made recommendations with knowledgeableness transition operators for R1 and also made recommendations with emotion and knowledgeableness transition operators for R2. We conducted this experiment with the aim of making the participants' state transition from state C to state D in Figure 1.

We recruited 296 Japanese participants for experiment 4, and 246 remained after noise exclusion. There were 98 males and 148 females, and they were aged between 16 and 66, for an average of 39.2 (*SD* = 10.4).

Table 7 shows the result of experiment 4. We conducted a Wilcoxon signed-rank test for these data. The PA significantly increased (*p* < 0.01), and the KS significantly decreased (*p* < 0.01). The ISS significantly increased (*p* < 0.01).

**Table 7:** Result of experiment 4.

**Scale**	**R1 average (SD)**	**R2 average (SD)**	**Increased pair**	**Decreased pair**	***p***
PA	18.01 (6.25)	19.22 (7.02)	125	90	0.0008
KS	4.26 (1.18)	4.01 (1.20)	79	126	0.0013
ISS	55.29 (16.98)	62.84 (20.96)	156	82	0.0000

We conducted the same test for the result of female participants. As a result, PA significantly increased (*p* = 0.008), KS significantly decreased (*p* = 0.000) and ISS significantly increased (*p* = 0.000). Also, we conducted the same test for the result of male participants. As a result, PA significantly increased (*p* = 0.036), KS non-significantly increased (*p* = 0.666) and ISS significantly increased (*p* = 0.000).

## 5. Discussion

### 5.1. Effect of Transition Operators

In experiments 1 and 4, we can see the effect of the emotion transition operators. As shown in both Tables 4, 7, the PA significantly increased. Also, the ISS significantly increased. These results mean that the emotion transition operators had an effect as we hypothesized in both of these experiments.

In experiments 2 and 3, we can see the effect of the knowledgeableness transition operators. As shown in both Tables 5, 6, the KS significantly increased. Also, the ISS significantly increased. These results mean that the knowledgeableness transition operators had an effect as we hypothesized in both of these experiments.

In the four experiments, both the emotion and knowledgeableness transition operators increased the perceived trustworthiness of the PRVA. These results show the validity of our model. However, the KS significantly decreased in both experiments 1 and 4. This means that the emotion transition operators reduced the perceived knowledgeableness of the PRVA. There is a possibility that the smiling and cute gestures were received as a sign of a lack of knowledge or intelligence. However, the ISS significantly increased when the KS significantly decreased. This result suggests that the emotion transition operators have more of an effect on trust than the knowledgeableness operators.

Why the emotion transition operators reduced the perceived knowledgeableness of the PRVA is an important question. Our consideration is that the participants perceived the PRVA's smile as being awkward. In this research, we used arm gestures and eye movement as a part of the emotion transition operators. These were reported as cues to deception (DePaulo et al., 2003). Thus, the participants may have unconsciously felt that the PRVAs were deceptive.

This effect suggested that emotion and knowledgeableness perceived can interfere with each other. However, experiments 1 and 3 showed that we could increase the trust level again by executing the knowledgeableness transition operators after executing the emotion transition operators. This result showed that we can use the knowledgeableness transition operators to amplify the trustworthiness that has already increased by executing the emotion transition operators. Thus, these operators are effective regardless of whether there is interference or not.

Little has been reported on the interference between emotion and knowledgeableness perceived. However, some studies reported that emotion and knowledgeableness perceived are another factors of decision making. In elaboration likelihood model, the customers used two kinds of buying decision making route, central route and peripheral route (Petty and Cacioppo, 1986). In central route, the customers will make decision based on logical thinking. In peripheral route, the customers will make decision based on their feeling, impression and heuristics. In our experiments, emotion is associated with peripheral route and knowledgeableness perceived is associated with central route. Dual-process theory suggested that the humans use two kinds of system for decision making, system 1 and system 2 (Evans and Stanovich, 2013). System 1 will be used when the human make decision immediately by feeling and system 2 will be used when the human make decision by deep thinking. Emotion seem to be associated with system 1 and knowledgeableness perceived seem to be associated with system 2. These models suggested two difference route or process, emotional route and logical route, in decision making. The interference between emotion and knowledgeableness perceived might mean the interference between two route. However, prior works define that two routes works independently, not coherently. This problem is our future work.

### 5.2. Observed Internal Behavior Transitions of Participants

**Figure 5:**
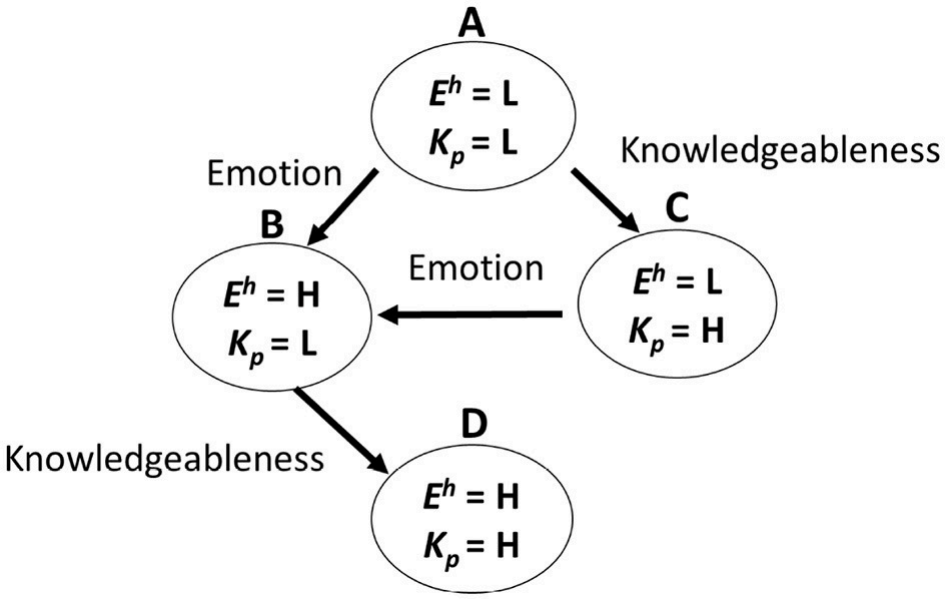
Observed transitions and effects of operators.

Figure 5 shows the internal behavior transitions observed in the four experiments. “Emotion” and “Knowledgeableness” means the transition operators that were executed.

In experiment 1, the participants' internal state transitioned from < *E*^*h*^ = L, *K*^*a*^ = L> to < *E*^*h*^ = H, *K*^*a*^ = L>. In experiment 2, it transitioned from < *E*^*h*^ = L, *K*^*a*^ = L> to < *E*^*h*^ = L, *K*^*a*^ = H >.

From the result of experiment 1, we concluded that the participants' state was < *E*^*h*^ = H, *K*^*a*^ = L> when they watched the PRVA with the emotion transition operators. Thus, we defined the participants' state as being < *E*^*h*^ = H, *K*^*a*^ = L> after R1 in experiment 3. The internal state transitioned from < *E*^*h*^ = H, *K*^*a*^ = L> to < *E*^*h*^ = H, *K*^*a*^ = H> after R2 in experiment 3. These results are suitable for our model.

From the result of experiment 2, we concluded that the participants' state was < *E*^*h*^ = L, *K*^*a*^ = H> when they watched the PRVA with the knowledgeableness transition operators. Thus, we defined the participants' state as being < *E*^*h*^ = L, *K*^*a*^ = H> after R1 in experiment 4. The internal state transitioned from < *E*^*h*^ = L, *K*^*a*^ = H> to < *E*^*h*^ = H, *K*^*a*^ = L> after R2 in experiment 3. This means the transition from C to B in Figure 1. In these experiments, we did not observe a transition from C to D. The reason seems to be that the effect of the emotion transition operators was too strong and interfered with the effect of the knowledgeableness transition operators. To observe the transition from C to D, we might adjust both operators.

The ISS significantly increased for all transitions. We defined the *T* (trust state) as transitioning from L to N in experiments 1 and 2. Also, in experiment 3, we defined *T* as transitioning from N to H. These results suggest that our hypothesis is correct. However, the ISS significantly increased also in experiment 4. According to our model, this result means that *T* transitioned from N to H. However, the internal state was < *E*^*h*^ = H, *K*^*a*^ = L> after R2 in experiment 4. In our model, *T* is N in this state. However, this is not a contradiction because we could increase the trustworthiness from this state as shown in experiment 3. It was shown that the < *E*^*h*^ = H, *K*^*a*^ = H> state has the most trustworthiness.

### 5.3. Limitations

This study has some limitations. First, we did not observe the transition from < *E*^*h*^ = L, *K*^*a*^ = H> to < *E*^*h*^ = H, *K*^*a*^ = H>. This suggested the limitation of our model or method. We guessed that this result was caused by the limitation of the transition operators. We expect that we can observe this transition when we will use other transition operators.

Second, we asked participants to fill out questionnaires after they watched each movie. To get a more accurate impression, we will use protocol data analysis. Also, to get a more objective result, we will use biological signals.

Third, there are gender difference in result. In experiment 1, 2, and 4, the both of female participants and male participants' ISS score significantly increased. In experiment 3, the male participants' ISS score did not significantly increase. Also, there are gender difference in some score at experiments. This result might be caused by gender difference in interacting virtual agents, or caused by small sample size.

Last, the generality of the results was not verified. Our model and method was effective for the PRVA; however, we cannot predicate that this method can be applied to other virtual agents. Cameron et al. suggested that a factor of robots' trustworthiness can come to the surface in a strong context experimental design (Cameron et al., 2015). In our experiment, the strength of context was not deeply considered.

## 6. Conclusion

We proposed a trust model and aimed at operating a user's trust toward PRVAs. Our original idea in this research is using two parameters to operate trust, a user's emotion and knowledgeableness perceived. Furthermore, we developed transition operators to make these parameters transition. Also, we conducted four experiments to verify this model with participants. In experiments 1 and 2, we verified the effect of the transition operators and successfully increased the participants' trust. In experiment 3, we executed knowledgeableness transition operators after emotion transition operators and observed the transition to a state that had the most trustworthiness. In experiment 4, emotion transition operators appeared after knowledgeableness transition operators, and we observed increased trustworthiness and decreased knowledgeableness perceived.

In these experiments, the knowledgeableness transition operators worked completely as expected, and the emotion transition operators definitely worked. The emotion transition operators restrained knowledgeableness perceived. This result suggested that these operators interfered with each other; however, this is not a big matter that increases trustworthiness. The emotion transition operators restrained the increase in trustworthiness regardless of the decrease in knowledgeableness perceived. Also, from the result of experiments 1 and 3, we discovered the most effective process to increase trustworthiness. When we executed the knowledgeableness transition operators after executing the emotion transition operators, we could cause the trust level to transition from L to H. This order is the most effective for making a user trust a PRVA.

These experiments have some limitations, and the interference between the emotion and knowledgeableness perceived is an unsolved problem. However, the transition model and transition operators suggested in this research can contribute to the design of PRVAs and other virtual agents.

## Author Contributions

TM conducted the experiments and analysis and drafted the manuscript with important contributions from SY. All authors participated in the review and revision of the manuscript and have approved the final manuscript to be published.

### Conflict of Interest Statement

The authors declare that the research was conducted in the absence of any commercial or financial relationships that could be construed as a potential conflict of interest.

## Footnotes

^1^https://crowdsourcing.yahoo.co.jp/

^2^http://www.mmdagent.jp/

^3^http://www.ah-soft.com/voiceroid/yukari/
